# 

**Published:** 1891-05

**Authors:** 


### CONTENTS FOR MAY, 1891.

ORIGINAL COMMUNICATIONS.

The Anatomical Position of the Caput Coli ; Deviations from the Normal Type. By THOMAS H. MANLEY, M. D .... 578
Notes on the Use of Koch's Lymph. By DR. E. THORNER. Translated by DR. JULIUS POHLMAN .... 582
A Year's Progress in Medicine. By P. M. WISE, M. D .... 587

SOCIETY PROCEEDINGS.

Philadelphia County Medical Society .... 592
Buffalo Obstetrical Society .... 600

CLINICAL REPORT.

Menthol in the Treatment of Erysipelas .... 606

PROGRESS IN MEDICAL SCIENCE.

Nervous Diseases .... 608
Surgery .... 611

SELECTION.

Gluten as a Food .... 615

CORRESPONDENCE.

Berlin Letter .... 620

EDITORIAL.

One of Those Mischievous Bills .... 623
The State Examining and Licensing Board Again .... 624
Niagara University Medical College .... 625
A String of Pearls from the *Post-Graduate* for April, 1891 .... 627
National Conference of State Medical Examining Boards .... 629
*The Journal of Gynecology* — Civil Service Examination for Physicians .... 622

PERSONAL.

Dr. L. S. McMurtry — Dr. C. C. Frederick — Dr. Nicholas Senn .... 630

SOCIETY NOTES.

American Medical Association .... 630
The Medical Association of Central New York .... 631

OBITUARY.

Dr. Charles T. Parkes .... 631

BOOK REVIEWS.

Wood's Medical and Surgical Monographs .... 631
A Dictionary of Practical Medicine .... 632
Twelve Lessons on the Structures of the Central Nervous System for Physicians and Surgeons .... 632
The Story of the Bacteria and their Relations to Health and Disease .... 633
The Year Book of Treatment for 1891 .... 633
Dust and Its Dangers .... 633
Professor Koch's Method to Cure Tuberculosis .... 634
Books and Pamphlets Received .... 634

